# Monte Carlo study of a new I‐125 brachytherapy prototype seed with a ceramic radionuclide carrier and radiographic marker

**DOI:** 10.1120/jacmp.v13i3.3741

**Published:** 2012-05-10

**Authors:** Lucas Paixão, Alessandro Facure, Ana Maria M. Santos, Adriano Márcio dos Santos, Suely Epsztein Grynberg

**Affiliations:** ^1^ Centro de Desenvolvimento da Tecnologia Nuclear Belo Horizonte/MG Brazil; ^2^ Comissão Nacional de Energia Nuclear Rio de Janeiro‐RJ Brazil

**Keywords:** Monte Carlo simulation, brachytherapy, iodine seeds

## Abstract

In prostate cancer treatment, there is an increasing interest in the permanent radioactive seeds implant technique. Currently, in Brazil, the seeds are imported with high prices, which prohibit their use in public hospitals. A ceramic matrix that can be used as a radioisotope carrier and radiographic marker was developed at our institution. The ceramic matrix is distinguished by the characteristic of maintaining the radioactive material uniformly distributed in its surface. In this work, Monte Carlo simulations were performed in order to assess the dose distributions generated by this prototype seed model, with the ceramic matrix encapsulated in titanium, in the same way as the commercial 6711 seed. The obtained data was assessed, as described in the TG‐43U1 report by the American Association of Physicists in Medicine, for two seed models: (1) the most used model 6711 source — for validation and comparison, and (2) for the prototype model with the ceramic matrix. The dosimetric parameters dose rate constant, Λ, radial dose function, $g_{L}(r)$, and anisotropy function, F(r,θ), were derived from simulations by the Monte Carlo method using the MCNP5 code. A Λ 0.992 (±2.33%) ${\text{cGyh}}^{-1}U^{-1}$ was found for the prototype model. In comparison with the 6711 model, a lower dose fall‐off on transverse axis was found, as well as a lower dose anisotropy for the radius r= 0.25 cm. In general, for all distances, the prototype seed model presents a slightly larger anisotropy between 0° ≤ Θ < 50° and anisotropy similar to the 6711 model for Θ ≥ 50°. The dosimetric characteristics of the prototype model presented in this study suggest that its use is feasible. Because of the model's characteristics, seeds of lower specific activity iodine might be necessary which, on the other hand, would help to reduce costs. However, it has to be emphasized that the proposed source is a prototype, and the required (AAPM prerequisites) experimental study and tolerance manufacturer values are pending for future studies.

PACS numbers: 87.53.Jw, 87.55.K

## I. INTRODUCTION

The modern permanent seed implant brachytherapy for the treatment of prostate cancer was introduced in the 1980s. This treatment modality is growing exponentially, mainly due to the favorable profile of normal tissues complications and time treatment (usually one day). With its excellent outcome, it has now become a standard treatment option, at least for prostate cancer low risk patients.^(^ ^1^ ^–^ ^3^ ^)^ The seeds used in the implants are usually made using 1 of 3 radioisotopes: ${\;}^{125}I$, ${\;}^{103}\text{Pd}$ and ${\;}^{131}\text{Cs}$.^(^ ^4^ ^)^ Several seed models exist on the market,^(^ ^5^ ^–^ ^6^ ^)^ but they all share three main physical characteristics: a radioisotope used for treatment, an encapsulation material, and a radiopaque material for imaging visualization.^(^ ^7^ ^)^ Several studies have been made to improve the dosimetric characteristics of the available seeds,^(^ ^7^ ^–^ ^14^ ^)^ and new designs and materials have been used in new seed models.^(^ ^15^ ^–^ ^18^ ^)^ Currently, in Brazil, permanent implants with ${\;}^{125}I$ seeds — the only seed model used in our country is the 6711 — are performed by a few medical institutions, mostly private ones, and the seeds are imported with high prices, which prohibits their use in public hospitals. Therefore, in order to reduce the financial cost of this treatment modality and to improve the dosimetric characteristics of the existing seeds, it is of great importance to have a national technology. A ceramic matrix that can be used as a radioisotope carrier and radiographic marker was developed at Nuclear Technology Development Center (CDTN) of the National Commission of Nuclear Energy (CNEN). The ceramic matrix is distinguished by the characteristic of maintaining the radioactive material uniformly distributed in its surface, besides being able to incorporate radioisotopes other than ${\;}^{125}I$. The purpose of this work is to characterize the dose distribution of a prototype seed model that has a ceramic matrix as a radioisotope carrier and a radiographic marker encapsulated in titanium, in the same way as the commercial 6711 seed, using the dose calculation described in the TG‐43U1 report by the American Association of Physicists in Medicine (AAPM).^(^ ^5^ ^)^ The dosimetric parameters dose rate constant, Λ, radial dose function, $g_{L}(r)$, and anisotropy function, F(r,θ), were derived from simulations using the Monte Carlo code MCNP5.

## II. MATERIALS AND METHODS

### A. Dose calculation formalism

In order perform the dosimetric characterization of the brachytherapy seeds, the update dose calculation protocol of the AAPM Task Group No. 43 (TG‐43U1)^(^ ^5^ ^)^ was employed, which is the current approach for dose calculation in treatment planning systems.^(^ ^19^ ^)^

### B. Brachytherapy sources

Two models of ${\;}^{125}I$ interstitial brachytherapy sources, the Oncura model 6711 (GE Healthcare, IL) and the prototype source (PS) with the ceramic matrix, were studied. Several articles have been published presenting the 6711 model dosimetric parameters, obtained through calculations or measurements, with the modifications introduced in the manufacturing process of the seed over the years. Currently their dosimetric parameters are still being published, mainly to meet the requirements of the TG‐43U1 that requires the reproduction of dose distributions for a published source model widely used, in order to publish dosimetric parameters of another seed model.^(^ ^12^ ^,^ ^14^ ^,^ ^17^ ^,^ ^20^ ^)^ Dolan et al.^(^ ^9^ ^)^ published the first dosimetric study that met the requirements of the TG‐43U1 protocol, both for dosimetry with TLDs and by Monte Carlo simulation. In the present work, the 6711 seed's dimensions are taken from that article. The source 6711 consists of a silver rod ($\rho =10.5\;g/{\text{cm}}^{3}$), covered along its entire length by a radioactive layer, encapsulated by a titanium cylinder ($\rho =4.54\;g/{\text{cm}}^{3}$). The radioactive layer is composed of a mixture of AgBr and AgI, present in the molecular ratio of 2.5:1, and its density is equal to $6.2\;g/{\text{cm}}^{3}$. The thickness of the radioactive layer is assumed to be 1.75 μm. The silver rod, which is the radiographic marker for this seed, has 2.8 mm long and 0.25 mm radius. Its ends are conical sections beveled at 45° and the end faces have a radius of 0.175 mm. The titanium encapsulation is 3.75 mm long, with thickness of 0.07 mm and outer diameter of 0.8 mm. The end welds are hemispheres with 0.375 mm radius. They observed that the inner surfaces of these welds were flat, convex and concave with equal frequency. So the seed was modeled with this surface as being flat. The total length of the seed is 4.55 mm.

**Figure 1:**
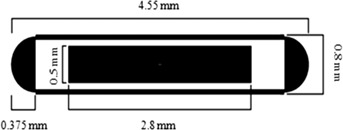
Cross‐sectional diagram of the prototype seed.

The prototype seed model (Fig. 1) has a high porous ceramic matrix that acts as radioactive material carrier and radiographic marker. The ceramic is made of 50% ${\text{SiO}}_{2}$ and 50% ${\text{WO}}_{3}$ with $\rho =3.59\;g/{\text{cm}}^{3}$. Its dimensions and encapsulation are the same of the 6711 source model except that the ceramic rod lacks the conical sections at its ends. The prototype seed with the same dimensions as the 6711 model would permit physicians to continue using the same type of implant procedures without the need of adopting new protocols or materials. It was assumed that the void within the Ti encapsulation of both seeds are filled with air at 40% humidity and 101.325 kPa ($\rho =0.00120\;g/{\text{cm}}^{3}$) with the composition recommended by the TG‐43U1.^(^ ^5^ ^)^

### C. Monte Carlo calculations

The dosimetric parameters Λ, $g_{L}(r)$ and F(r,θ) for both seed models were obtained by Monte Carlo simulations. The Monte Carlo N‐Particle code version 5 (MCNP5) was used in this study. MCNP5 is a general purpose Monte Carlo radiation transport code that allows for a coupled neutron–photon–electron transport calculation, in three dimensions, through complex geometries constructed by combinations of planes, spheres, cones, and cylinders.^(^ ^21^ ^)^ Due to the energy range of this study (< 40 keV), we can assume that there is charged particle equilibrium (CPE) and the collision kerma can be considered equal to the absorbed dose. Therefore, the MCNP F6 tally (track length estimator) is the best estimator for the determination of the collision kerma to derive TG‐43 dose parameters.^(^ ^22^ ^)^ Only photons were simulated and the detailed photon physics treatment includes photoelectric absorption, K and L shell fluorescence, Auger emission, coherent scattering with electron binding effects accounted for by form factors, and incoherent scattering. The MCNP5 default MCPLIB04 cross‐section library, which is based on the EPDL97 tables,^(^ ^23^ ^)^ and the ${\;}^{125}I$ spectrum from the TG‐43U1 were used in the simulations. The sources were modeled in the center of a water sphere ($\rho =0.998\;g/{\text{cm}}^{3}$) with 18.6 cm radius. To determine the dosimetric parameters, the scoring cells where the quantities of interest are computed were projected in a way that their volume does not affect the value of the dosimetric parameter being estimated, as recommended by the TG‐43U1.^(^ ^5^ ^)^ In the literature, various geometries are used for these cells, such as cones intercepted by spherical shells,^(^ ^11^ ^)^ cylindrical rings,^(^ ^24^ ^)^ toroids,^(^ ^13^ ^)^ voxels^(^ ^7^ ^,^ ^12^ ^,^ ^25^ ^)^. In some publications the geometries used are not mentioned.^(^ ^10^ ^,^ ^14^ ^,^ ^26^ ^)^ To determine $g_{L}(r)$ and F(r,θ), water spheres centered in the (r,Θ) points were used. The Θ points of F(r,θ) range from 0° to 90° in increments of 5°. The r points of F(r,θ) and $g_{L}(r)$ ranges from 0.25 to 10 cm (0.25, 0.5, 1, 2, 3, 4, 5, 6, 7, 7.5 and 10 cm). The scoring cells radii $\left(r_{\text{sc}}\right)$ vary as follows: for r≤1cm, $r_{\text{sc}}=6.2\times 10^{-3}\text{cm}$; for 1<r≤5cm, $r_{\text{sc}}=3.1\times 10^{-2}\text{cm}$; for 5<r≤10cm, $r_{\text{sc}}=6.2\times 10^{-2}\text{cm}$. The radii of the water sphere and the scoring cells are based on the rectilinear water phantom and voxels of the work of Taylor and Rogers.^(^ ^12^ ^)^ The number of histories simulated was $4\;\times \;10^{10}$ for all studied cases, which represents a statistical error less than 2% (one standard deviation uncertainty, 1σ) on the calculated dosimetry parameters at a distance of 10 cm from the source. For the determination of Λ, the seed was considered as being placed in the center of a vacuum sphere with 15 cm radius. In this simulation, an energy cutoff of δ = 5 keV was used to suppress the characteristic X‐rays generated in the Ti encapsulation as recommended by TG‐43U1. The scoring cell should have similar dimensions to the solid angle subtended by the primary collimator of the WAFAC (Wide‐angle Free‐air Chamber) ionization chamber of NIST.^(^ ^5^ ^)^ Therefore, in this step, a volume element of $2.7\;\times \;2.7\;\times \;0.05\;{\text{cm}}^{3}$, located at distance d = 10 cm in transverse axis of the source was considered as scoring cell.^(^ ^12^ ^)^ To determine the air kerma in the cell, the MCNP *F4 tally was used for determining the energy fluence in the cell. To determine the air kerma, the MCNP's DE/DF card was considered, with the mass energy transfer coefficients of air being the parameters, to assess the air kerma values. The mass energy transfer coefficients were calculated using the composition of air with 40% relative humidity of TG‐43U1 and the coefficients of Seltzer.^(^ ^27^ ^)^ This method for derivation of dose rate constant is consistent with other works.^(^ ^10^ ^,^ ^11^ ^,^ ^20^ ^)^ The simulations were performed with $5\;\times \;10^{8}$ histories.

### D. Geometric uncertainty

According to TG43U1, the total percentage uncertain, $\sigma_{Y\%}$ of each dosimetric parameter Y (Λ, $g_{L}(r)$, F(r, Θ)) is considered as being composed by three main sources: the uncertainty of type B due to the cross section uncertainty $\left(\%\sigma_{Y/\mu }\right)$, type B uncertainties due to the geometry of the seed $\left(\%\sigma_{Y/\text{Geo}}\right)$, and type A uncertainty, inherent in the Monte Carlo technique $\left(\%\sigma_{Y/s}\right)$. Monte Carlo simulations were conducted to quantify the influence of geometrical uncertainties in the model 6711 seed dosimetric parameters, according to the methodology proposed by Dolan et al.^(^ ^9^ ^)^ The geometric uncertainty in this study consists of the following geometric parameters, ${\text{geo}}_{i}$: (1) vertical position of the Ag wire inside the encapsulement (4 mm displacement), (2) thickness of the material at the end of the seed (±0.12 mm), and (3) radioactive layer thickness (1.0 to 2.5 mM), (4) thickness of the Ti encapsulation (±0.01 mm), (5) diameter of the silver wire (60 to 80% of its diameter). For each of the dosimetric parameters, 10 simulations were performed. For example, to estimate the uncertainty of $g_{L}(r)$ for each geometric uncertainty geoi, i varying from 1 to 5, two simulations were performed: for the first simulation, the dose scoring in the cells was determined with the geometric parameter in the maximum value geoi=(geoi) max, and the second simulation with geoi=(geoi) min, with all other geometrical parameters with their nominal values.^(^ ^9^ ^)^ After making the simulation and uncertainty estimates for all other geometrical parameters, the geometric uncertainties are combined using the Law of Propagation of Uncertainty (LPU) to estimate the total uncertainty of the parameter Y uncertainty due to geometric σY/Geo. Finally, the total geometric uncertainty is combined with other sources of uncertainty (MC statistics, cross sections etc.) and presented in a table. All the uncertainties estimated in this paper are standard uncertainties, with a coverage factor of one (k = 1), with a confidence level of approximately 68%.

## III. RESULTS & DISCUSSION

### A. Dose‐rate constant

The dose‐rate constant calculated values are shown in Table 1, together with the values determined by other authors. All values presented are derived for a similar geometry to the WAFAC ionization chamber. The value of the 6711 model dose‐rate constant calculated in this study agrees with the range of uncertainties of calculated and measured values by Dolan et al.^(^ ^9^ ^)^ and the consensus value recommended by TG‐43U1 protocol.^(^ ^5^ ^)^ The relative percentage difference from the value calculated by Taylor and Rogers^(^ ^12^ ^)^ is 2.30%. The uncertainty of Λ calculated by them only takes into account statistical uncertainties of the MC calculation, without assessing other sources of uncertainties such as, for example, geometric uncertainties. The uncertainty due to energy spectrum, seed geometry, and MC statistics are 0.1%, 0.03% and 0.03%, respectively. The component that represents most impact on the uncertainties is the cross section, 1.2%. The Λ uncertainty calculated in this study, 1.2%, is below the generic value of the value recommended by TG‐43U1 for this parameter of 3% when determined by MC simulations,^(^ ^5^ ^)^ and of the same order of magnitude that uncertainty estimated by Dolan et al.^(^ ^9^ ^)^

**Table 1:** Dose‐rate constant values for the model 6711 and the prototype seed (PS) with their respective estimated combined uncertainties.

*Author*	*Source Model*	*Method*	$\Lambda (cGyh^{-1}U^{-1})$
This study	6711	MCNP5	0.945 ± 0.011
This study	PS	MCNP5	0.992 ± 0.023
Dolan *et al*.^(^ ^9^ ^)^	6711	PTRAN	0.942 ± 0.017
Dolan *et al*.^(^ ^9^ ^)^	6711	TLD	0.971 ± 0.059
Taylor and Rogers^(^ ^12^ ^)^	6711	EGSnrc	0.924 ± 0.002
TG‐43U1 consensus value	6711	MC + Exp	0.965 ± 0.046

Table 2 presents the uncertainty analysis for the determination of the dose rate constant of the PS model, as recommended by the TG‐43U1 protocol. The geometric uncertainty used in the calculation of total uncertainty of the prototype seed (PS) dose‐rate constant is equal to 2%. This value was employed because the seed is still in project phase and, according to the TG‐43U1 protocol,^(^ ^5^ ^)^ this is a conservative and reasonable estimate for $\%\sigma_{\wedge /\text{Geo}}$. The other components of uncertainty (cross sections and energy spectrum) are the same as the ones for the 6711 model, except the Monte Carlo uncertainty that was of 0.05%. The difference between the values of the dose‐rate constants of the models PS and 6711 is 5%. Taking into account the uncertainty intervals, we can say that the dose‐rate constants of the two models are different, with the prototype seed model depositing a higher dose at the reference point for the same air kerma strength of the source. It is believed that these differences can be attributed to the higher amount of iodine that can be incorporated in the prototype seed.

**Table 2:** Uncertainties associated with the evaluation of PS model's dose‐rate constant.

	*Monte Carlo Uncertainties*	
*Component*	*Type A*	*Type B*
Cross sections^(^ ^23^ ^)^		1.20%
Energy spectrum^(^ ^5^ ^)^		0.10%
Seed geometry		2.00%
Monte Carlo statistics	0.05%	
Quadratic sum	0.05%	2.33%
Total expanded uncertainty (k =1)	2.34%	

### B. Radial dose function

**Figure 2:**
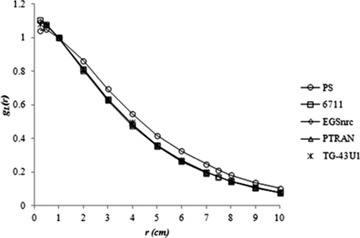
Radial dose functions calculated in this study and by other authors.

The values of radial dose function $g_{L}(r)$ for the model 6711 and the prototype seed (PS) calculated in this study, as well as the values determined by other authors, are presented in Fig. 2. All calculated values of 6711 model radial dose function have relative percentage difference of less than 1% when compared with values calculated by Taylor and Rogers.^(^ ^12^ ^)^ The largest difference, in comparison with the values determined by Dolan et al.,^(^ ^9^ ^)^ was 2.15%. Regarding the values recommended by TG‐43U1,^(^ ^5^ ^)^ the largest difference was 3.1% for r = 10 cm.

The radial dose function of the prototype seed shows a slightly lower dose fall‐off in the transverse axis when compared with the 6711 model, for r > 1 cm, and a lower dose deposition for r ≤ 1 cm. But, when we calculate the dose rate distribution (cGy/h) with the same $S_{K}$, the dose rate distribution in the transverse axis of both sources are very similar. This result illustrates that the scattering and attenuation of photons in the transverse axis of the prototype source is not very diverse from a commercial source.

### C. Anisotropy function

**Figure 3:**
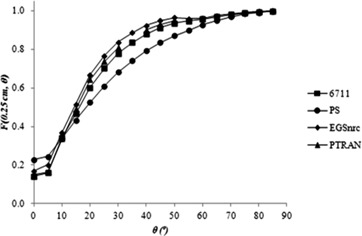
2D anisotropy function of the 6711 model and prototype seed (PS) model for r = 0.25 cm.

**Figure 4:**
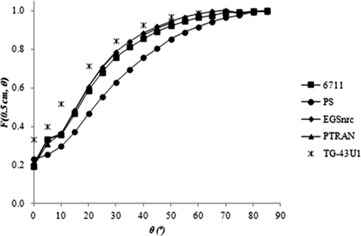
2D anisotropy function of the 6711 model and prototype seed (PS) model for r = 0.5 cm.

**Figure 5:**
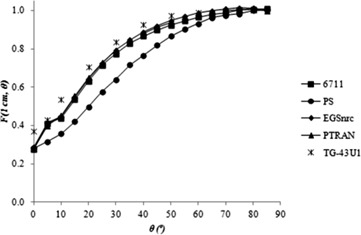
2D anisotropy function of the 6711 model and prototype seed (PS) model for r = 1 cm.

Figures 3, 4 and 5 show the anisotropy functions for 6711 models, calculated in this study and by other authors, and for the PS seed, to 0.25≤r≤1 cm. According to the TG‐43U1 protocol,^(^ ^5^ ^)^ when comparing anisotropy function values, the difference between the data is typically 5% or up to a maximum of 9% for Θ > 30°. For Θ ≤ 30°, the differences are larger (typically 10% reaching a difference of about 17%) and can be attributed to: the effect of the volume of the cells used for estimating the dose in (r,Θ) points; the high‐dose gradient near the longitudinal axis of the source, and geometric uncertainties. The comparison of the values of the 2D anisotropy function for the 6711 seed model calculated in this study with the results produced by Dolan et al.^(^ ^9^ ^)^ and Taylor and Rogers,^(^ ^12^ ^)^ typically range from 0.1% to 4.5% for most of the (r,Θ) points. The largest discrepancies found were 42% for F(0.5 cm, 0°), compared to the recommended value by TG‐43U1, and 20.7% for F(0.25 cm, 5°), compared with the calculated value Taylor and Rogers.^(^ ^12^ ^)^

The values for the prototype seed model are shown in Table 3. For a distance r = 0.25 cm from the source, the prototype seed model has a lower dose anisotropy than the 6711 model. Another important feature of the prototype model is that its anisotropy curves are smoother than the 6711 model curves, for Θ ≤ 10°. In general, for all distances, the PS model presents a slightly larger anisotropy between 0° ≤ Θ < 50° and anisotropy similar to the 6711 model, for Θ ≥ 50°. These differences can be attributed to the different distributions of radioactive material inside the source, since this aspect impacts on the anisotropy.

**Table 3:** 2D Anisotropy function values for the prototype seed model calculated in this study.

	*r (cm)*												
Θ(°)	0.25	0.5	1	2	3	4	5	6	7	7.5	8	9	10
0	0.227	0.230	0.282	0.363	0.410	0.446	0.475	0.477	0.488	0.502	0.502	0.515	0.505
5	0.244	0.254	0.315	0.386	0.432	0.461	0.491	0.492	0.507	0.522	0.524	0.534	0.534
10	0.339	0.296	0.357	0.423	0.466	0.494	0.522	0.528	0.533	0.550	0.557	0.562	0.562
15	0.432	0.372	0.419	0.478	0.519	0.544	0.575	0.571	0.584	0.585	0.597	0.591	0.590
20	0.525	0.466	0.498	0.544	0.580	0.605	0.612	0.619	0.624	0.631	0.648	0.670	0.639
25	0.609	0.552	0.577	0.613	0.642	0.653	0.684	0.670	0.679	0.681	0.695	0.705	0.688
30	0.681	0.629	0.639	0.672	0.695	0.709	0.730	0.719	0.725	0.725	0.741	0.707	0.742
35	0.741	0.697	0.716	0.731	0.750	0.759	0.778	0.766	0.771	0.774	0.779	0.774	0.763
40	0.793	0.756	0.766	0.781	0.803	0.799	0.812	0.805	0.795	0.809	0.815	0.806	0.795
45	0.835	0.805	0.820	0.828	0.836	0.844	0.844	0.840	0.837	0.859	0.852	0.858	0.846
50	0.870	0.852	0.866	0.864	0.878	0.885	0.886	0.876	0.871	0.888	0.888	0.894	0.873
55	0.898	0.888	0.901	0.903	0.914	0.920	0.933	0.913	0.914	0.920	0.912	0.921	0.891
60	0.929	0.916	0.930	0.928	0.934	0.938	0.939	0.928	0.933	0.944	0.937	0.938	0.922
65	0.950	0.944	0.964	0.946	0.961	0.962	0.972	0.949	0.960	0.962	0.955	0.960	0.959
70	0.969	0.965	0.975	0.969	0.979	0.981	0.983	0.972	0.970	0.978	0.960	0.978	0.942
75	0.984	0.977	0.983	0.984	0.991	0.995	1.003	0.987	0.984	0.981	0.993	0.969	0.982
80	0.990	0.994	1.000	0.993	1.001	0.998	1.006	0.995	0.992	0.986	0.999	0.971	0.986
85	0.998	0.997	1.004	0.998	1.006	1.006	1.028	1.000	0.996	1.002	0.998	0.992	1.008

## IV. CONCLUSIONS

The TG‐43 dosimetric parameters of a prototype seed model that has a ceramic matrix as a radioisotope carrier and a radiographic marker encapsulated in titanium were determined by the Monte Carlo method. The obtained dose‐rate constant indicates a probability of a higher dose deposition at the reference point, for the same air kerma strength of the source, in comparison the 6711 seed model. The radial dose function of the prototype seed shows a lower dose fall‐off in the transverse axis when compared with the 6711 model, for r> 1 cm, and presents a slightly larger anisotropy between 0° ≤ Θ < 50° and an anisotropy similar to the 6711 model for Θ ≥ 50°. The dosimetric characteristics of the ceramic matrix seed presented in this study suggest that its use is feasible. Based on these results, a newly developed prototype seed might be an alternative to other I‐125 sources. However, according to the Low‐energy Interstitial Brachytherapy Dosimetry subcommittee of the AAPM Radiation Therapy Committee (LIBD), an independent experimental study must be performed prior to clinical utilization. It has to be emphasized that the proposed source is a prototype, and that the required (AAPM Prerequisites) experimental study and tolerance manufacturer values are pending for future studies.

## ACKNOWLEDGMENTS

The authors would like to thank the Comissão Nacional de Energia Nuclear for financial support.
